# Rapid identification of chloroquine and atovaquone drug resistance in *Plasmodium falciparum *using high-resolution melt polymerase chain reaction

**DOI:** 10.1186/1475-2875-9-134

**Published:** 2010-05-21

**Authors:** Linda Seo Hwee Gan, Jin Phang Loh

**Affiliations:** 1 Population Genetics Laboratory, Defence Medical & Environmental Research Institute, DSO National Laboratories, 27 Medical Drive, 117510, Singapore; 2 Detection and Diagnostics Laboratory, Defence Medical & Environmental Research Institute, DSO National Laboratories, 27 Medical Drive, 117510, Singapore

## Abstract

**Background:**

Drug resistance determination for *Plasmodium falciparum *infections are important to determining the type of treatment to be given. Besides *in vivo *experiments, molecular methods, such as sequencing and PCR, are now increasingly being used. Here a cheaper alternative to sequencing or the use of multiplex 5'nuclease PCR assay for detection and differentiation of drug resistance haplotypes for chloroquine and atovaquone using polymerase chain reaction-high resolution melt (PCR-HRM) is reported.

**Methods:**

Separate PCR-HRM assays were designed for the detection and differentiation of chloroquine and atovaquone drug resistance haplotypes in *P. falciparum*. PCR was conducted on a thermal cycler and melt curves generated using a LightScanner. These were tested against reference strains of *P. falciparum *from MR4 as well as 53 local isolates.

**Results:**

The PCR-HRM assays are able to detect and differentiate between the various haplotypes consistently. These assays can also be used to detect new variants.

**Conclusions:**

PCR-HRM is an inexpensive option for the determination of drug resistance profile in *P. falciparum *and will see increasing use as an alternative to sequencing and 5'nuclease PCR assays in reference laboratories or once PCR systems that are able to conduct HRM become commonplace.

## Background

The molecular basis of chloroquine resistance (CQR) in *Plasmodium falciparum *is still relatively unclear, and the association of point mutations in different genes with CQR has been largely studied in the last decade. In 2000, the *pfcrt *gene was identified, which appears to play a crucial role in CQR. A lysine to threonine change at position 76 (K76T), which was subsequently found in every *in vitro *CQR parasite from around the world, was identified as an important mutation associated with CQR [1]. Although this mutation is not the sole requirement for determining of CQR, the absence of the K76T mutation is highly predictive of CQ sensitivity *in vitro *and CQ efficacy *in vivo *[2].

Similarly, the molecular basis of atovaquone resistance in *P. falciparum *has been suggested to be due to mutations in the cytochrome *bc1 *gene of the parasite mitochondrial genome, which prevents binding of atovaquone to the cytochrome [3]. In particular, mutations at codon 268 are associated with atovaquone/proguanil treatment failure *in vivo *and can be used as possible resistance markers. Both the amino acid changes, Y268N and Y268S, result in extremely high increase in resistance levels [4]. There are also recent reports of Y268C mutants that are also highly resistant to atovaquone [5].

One of the most common methods for single nucleotide polymorphism (SNP) detection is the conduct of a polymerase chain reaction (PCR) flanking the mutation site, followed by sequencing for confirmation [6]. This process is however both laborious and expensive. Following the development of real-time PCR and relevant fluorescence detection systems, SNP detection became possible using fluorescent probe chemistries, such as the 5'nuclease PCR assay and fluorescent resonance energy transfer (FRET) PCR. These assays confer specificity while eliminating the need for confirmation *via *sequencing. Subsequently, PCR assays for the detection of chloroquine resistance based on these technologies were developed and widely used [7-9]. However, the use of fluorescent probes is limited to single SNP detection per probe and prior knowledge of the SNP location to design the detection probes. Thus, this method is relatively expensive especially in a multiplex PCR where several different fluorescent probes are required. In addition, there may be more SNPs at a particular locus than combination fluorescent probes that can be utilized. Hence, high resolution melting (HRM) analysis offers potential as a solution and here a PCR-HRM method for distinguishing between wild type and drug-resistant *P. falciparum *strains in a single reaction without the need for multiple fluorescent probes is presented.

The utility of HRM analysis for detection of point mutations in *P. falciparum *genes associated with drug resistance has been demonstrated earlier [10]. The technique employed, however, is heavily reliant on the resolution power of the equipment as differences in melting temperature (Tm) between wild-type and mutant-type isolates are less than 2°C. Furthermore, as the size of the amplicon increases, the ability to detect small Tm difference decreases, thereby imposing a restriction on PCR primer design. Here, asymmetric PCRs were developed free from amplicon size restriction as the haplotype discrimination depends on a short (20 - 35 nucleotides), unlabeled 3'-blocked probe and LC Green Plus ^® ^fluorescent dye. The assay is based on differences in melting temperature between perfect matches and mismatches under the probes and has been shown to be useful for SNP genotyping in genetic studies [11].

## Methods

### Materials

DNA from reference strains of *P. falciparum *(harbouring the known chloroquine resistance profiles) were provided by Malaria Research and Reference Reagent Resource Center (MR4) USA. These include MRA-152G, MRA-155G, MRA-156G, MRA-175G, MRA-202G and MRA-387G. DNA from these strains were used at a concentration of 1 pg/μl. Synthetic constructs were made for the atovaquone-resistant profile types Y268N and Y268S and were used at a concentration of 1 fmol/μl. A *P. falciparum *blood sample with atovaquone-resistant profile Y268C was obtained from Dr Colin Sutherland, London School of Hygiene and Tropical Medicine, UK. 53 other isolates of *P. falciparum *used in this study were part of a collection from DSO National Laboratories which were previously checked *via *PCR for mono-infection.

### DNA extraction

DNA was extracted from *P. falciparum *positive blood samples (parasitaemia greater than 0.05%) using the QIAamp DNA Mini Kit (Qiagen Inc). 200 μl of blood sample was used for extraction and DNA eluted in 100 μl of elution buffer.

### PCR-HRM for the detection of chloroquine resistance

Primers used were previously described [1] and probes were designed using Primer 3 software [12]. The PCR was set up in a final volume of 10 μl containing 1 μl of DNA, 75 nM of CRTD1 primer (5'-TGTGCTCATGTGTTTAAACTT-3'), 375 nM of CRTD2 primer (5'-CAAAACTATAGTTACCAATTTTG-3'), 500 nM of CRTWT probe (5'-TGTATGTGTAATGAATAAAATTTTTGC-phosphate-3'), 375 μM dNTP mix, 1× reaction buffer, 1× LC Green Plus (Idaho Technology Inc., USA), 6.875 mM MgCl_2 _and 0.5 U of Platinum Taq polymerase (Invitrogen Corp). After overlaying the PCR mix with 20 μl mineral oil, the following conditions were used: 95°C for 6 min; 60 cycles of 95°C for 15 s and 55°C for 1 min; followed by 94°C for 30 s and 25°C for 30 s for heteroduplex formation and 15°C for storage. This is followed by melt curve analysis on the LightScanner (Idaho Technology Inc., USA).

### Real-time PCR for the detection of chloroquine resistance

The chloroquine resistance haplotypes for all *P. falciparum *isolates were determined using the multiplex real-time PCR assay developed by Sutherland *et al *[8].

### PCR-HRM for the detection of atovaquone resistance

Primers and probes were designed using Primer 3 software. The PCR was set up in a final volume of 10 μl containing 1 μl of DNA, 50 nM of cytbF primer (5'-CACATCCTGATAATGCTATCG-3'), 250 nM of cytbR primer (5'-AGCTGGTTTACTTGGAACAG-3'), 500 nM of WT probe (5'-GGTACTTTCTACCATTTTATGCAATG-phosphate-3'), 100 μM dNTP mix, 1× reaction buffer, 1× LC Green Plus, 1.5 mM MgCl_2 _and 0.5 U of Platinum Taq polymerase. After overlaying the PCR mix with 20 μl mineral oil, the following conditions were used: 95°C for 6 min; 60 cycles of 94°C for 10 s, 55°C for 10 s and 72°C for 10 s; 72°C for 3 min, followed by 94°C for 30 s and 25°C for 30 s for heteroduplex formation and 15°C for storage. This is followed by melt curve analysis on the LightScanner (Idaho Technology Inc., USA).

### PCR-RFLP for the detection of atovaquone resistance

The atovaquone resistance haplotypes for all *P. falciparum *isolates were determined using the PCR-RFLP method developed by Schwöbel *et al *[4].

### Sensitivity of HRM assay in single and mixed strains reactions

In order to determine HRM assay sensitivity, serial dilutions of DNA (10^-1 ^to 10^-5 ^ng/μl) were tested. The *pfcrt *HRM assay was carried out on three different reference strains, MRA-102G (CVMNK haplotype), MRA-152G (SVMNT haplotype) and MRA-150G (CVIET haplotype) in duplicates. The same procedure was also performed on the atovaquone HRM assay but only with MRA-102G wild type reference strain.

The ability of the method to detect a minority haplotype in a mixture of wild- and mutant-type *pfcrt *alleles was also assessed. DNA concentration of both MRA-102G (CVMNK) and MRA-150G (CVIET) reference strains were adjusted to 0.1 ng/μl and combined to have the following CVMNK/CVIET ratio: 10:90, 30:70, 50:50, 70:30, 90:10.

### DNA Sequencing

Sanger's dideoxy sequencing was carried out on Exo-SAP purified PCR products using Big Dye Terminator v3.1 chemistry and electrophorezed on the Applied Biosystem 3730 Genetic Analyzer (Applied Biosystems, USA).

## Results

### PCR-HRM for the detection of chloroquine resistance

**Figure 1:**
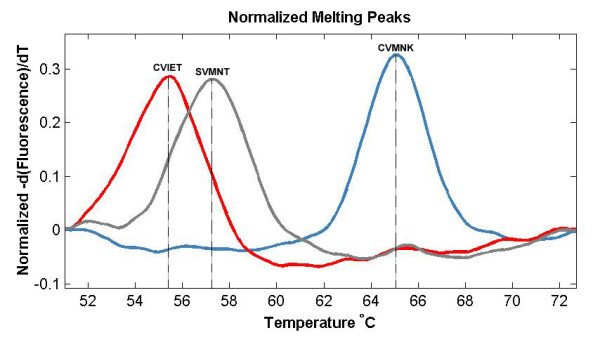
**High resolution melt profile of the 3 *pfcrt *haplotypes**.

**Figure 2:**
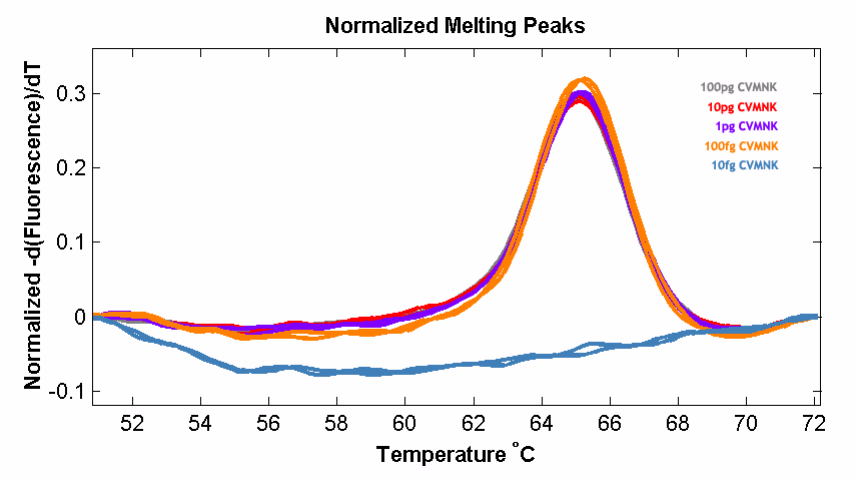
**High resolution melt profile for the *pfcrt *sensitivity assay using MRA-102G**.

**Figure 3:**
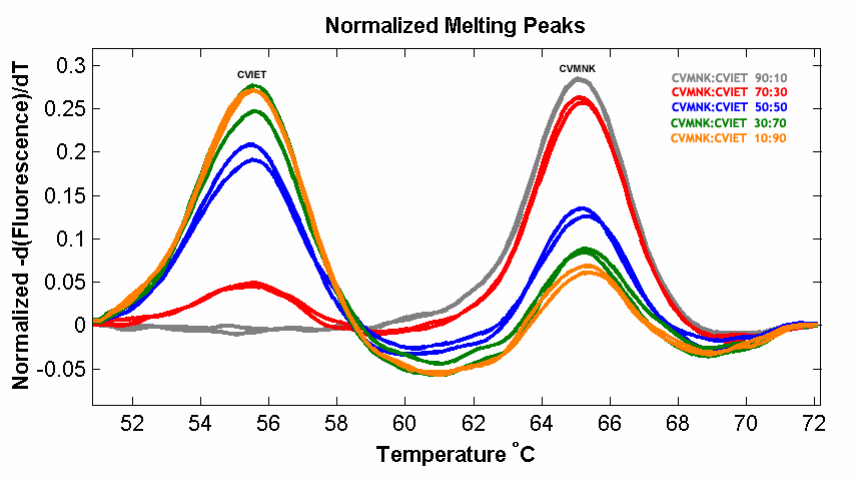
**High resolution melt profile of different proportions of CVMNK:CVIET mixes to assess the detection threshold of the minority haplotype**.

**Figure 4:**
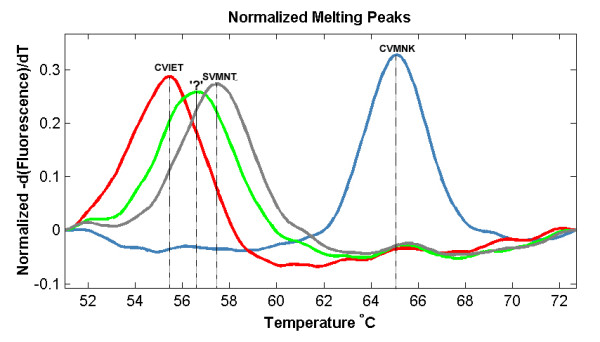
**High resolution melt profile of the 'unknown' haplotype with respect to the 3 common *pfcrt *haplotypes**.

**Figure 5:**
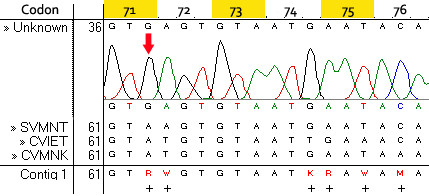
**"Unknown" haplotype has a point mutation (A->G) in codon 71 just upstream of the SVMNT signature**.

The results show that the wild type haplotype (CVMNK) could be easily differentiated from the mutant resistant haplotypes (CVIET and SVMNT) since the difference in Tm is more than 7°C (Figure 1). The difference between CVIET and SVMNT haplotypes is only 2°C but this has been tested to be reproducible and consistent. The method had a detection threshold of 10^-4 ^ng/μl for the wild type (CVMNK) (Figure 2) and SVMNT haplotype, while the detection threshold for CVIET haplotype was higher at 10^-3 ^ng/μl. A similar trend for sensitivity was observed when detecting minority haplotype in mixed-alleles samples; only 10% of wild-type haplotype is required to be present in a mixture in order to be detected by the HRM assay, whereas at least 30% of the CVIET haplotype is required in a mixture before being detected due to the assay's lower sensitivity for the CVIET haplotype (Figure 3). The developed method was tested against 53 *P. falciparum *strains that have previously been typed using a real-time PCR method developed by Sutherland *et al *[8]. Of the 53 isolates tested, five had the CVMNK haplotype, 18 had the CVIET haplotype and the remaining 30 isolates had the SVMNT haplotype. 96.2% concordance was achieved between the two methods because two of the 30 SVMNT isolates gave a Tm peak that was 1°C higher than that of the CVIET haplotype (Figure 4). Although real-time PCR typed these two strains as SVMNT haplotypes, sequencing of the PCR product confirmed that they are of the SVMNT haplotype but with an additional "A" to "G" point mutation in codon 71 just upstream of codon 72 (Figure 5). Whether this mutation has an effect on chloroquine sensitivity of these two isolates was not explored further in this study.

### PCR-HRM for the detection of atovaquone resistance

**Figure 6:**
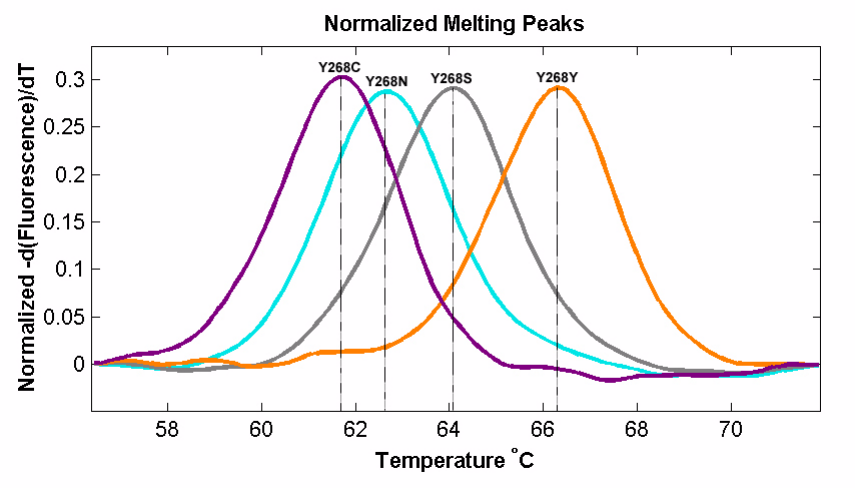
**High resolution melt profile of the 4 haplotypes for detection of atovaquone resistance using the cytB protocol**.

**Figure 7:**
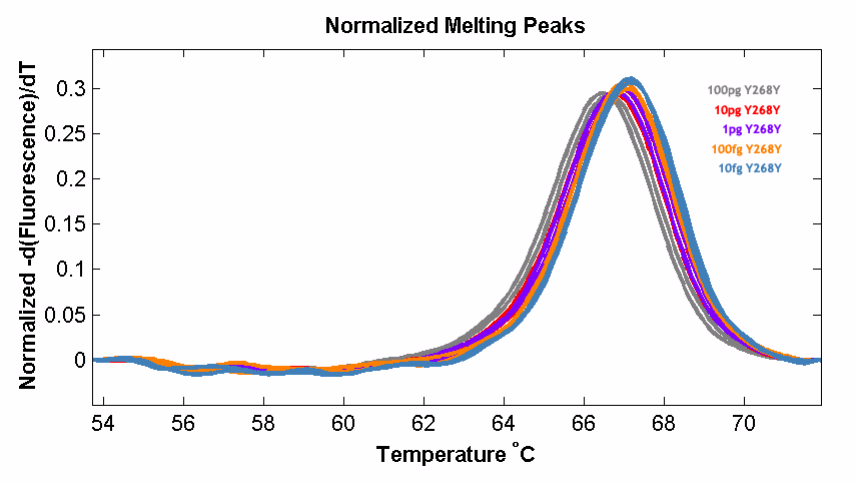
**High resolution melt profile for the cytB sensitivity assay using MRA-102G**.

The results show that the wild type haplotype (Y268Y) could also be easily differentiated from the mutant resistant haplotypes (Y268N, Y268S and Y268C) since the difference in Tm is more than 6°C (Figure 6). The Tm differences between the mutant haplotypes however are much smaller at between 1 to 1.5°C but have also shown to be reproducible and consistent. The detection threshold for wild type haplotype is higher compared to that for the *pfcrt *PCR-HRM at 10^-5 ^ng/ul (Figure 7). Sensitivity for other haplotypes was not tested due to lack of isolates for testing. The same 53 *P. falciparum *strains were tested and compared against their haplotypes determined using PCR-RFLP according to the method developed by Schwobel *et al *[4]. In this case, all 53 isolates had the wild type haplotype (Y268Y). 100% concordance was achieved between the two techniques.

## Discussion

The PCR-HRM technique developed here is relatively inexpensive compared to the use of multiple fluorescent probes or sequencing [13]. It provides a quick way to differentiate between drug-resistant strains and drug-sensitive strains using only a single unlabeled probe in an asymmetric PCR followed by a melt curve which is easily done without further chemistries. This technique is an improvement on the assay developed by Andriantsoanirina *et al *[10], as the use of the unlabeled probe allows for very clear distinction between wild-type and mutant type strains. Difficulties may be encountered in determining the exact resistant haplotype due to the small difference in Tm if PCR conditions and reagents used are not optimal or there is a lack of controls for each haplotype in the run. However, determination of drug resistance on a qualitative scale (i.e. sensitive or drug resistant) is relatively easy with this assay due to the larger Tm differences between wild type (sensitive) haplotype and resistant haplotypes.

Its ease of use is especially observed for detection of atovaquone resistance where alternatives currently available are sequencing and PCR-RFLP [4]. Development of a multiplex 5'nuclease PCR assay to differentiate between the four haplotypes is not impossible but will likely require the use of multiple Lock-nucleic acid (LNA) or Minor grove binding (MGB) fluorescent-tagged probes which adds to the cost of the assay.

In addition, the use of HRM allows for the detection of new variants from the wild type, which could potentially be drug-resistant as well. It is predicted that the PCR-HRM method developed for *pfcrt *will be useful for the detection and differentiation of CVMNT, CVIKT and SVIET haplotypes discovered in Papua New Guinea [14] from wild type as well. This is further exemplified in the detection of two *P. falciparum *strains harbouring a point mutation just upstream of the variable region in *pfcrt *gene and confirmed by sequencing. The importance of this mutation is not known but it would have gone unnoticed if only the 5'nuclease PCR assay was used for detection purposes.

In this study, two systems were used in combination (conventional thermal cycler and the LightScanner) to generate the required result. There are now systems in the market that can do PCR and HRM together such as the Roche Lightcycler 480, Applied Biosystems ABI 7500 Fast PCR system as well as the Qiagen Rotor-Gene 6000 and it is expected that as such systems become more commonplace, it will provide a better option for seamless operation. However this needs to be tested to ensure that the HRM is of sufficient resolution power and robust enough to differentiate between mutant haplotypes consistently.

## Conflict of interests

The authors declare that they have no competing interests.

## Authors' contributions

LSHG carried out the PCR-HRM studies and drafted the manuscript. JPL carried out the 5'nuclease PCR and PCR-RFLP studies. JPL conceived of the study, participated in its design and coordination as well as revision of the manuscript. All authors read and approved the final manuscript.
